# Ferulic acid ameliorates bisphenol A (BPA)-induced Alzheimer’s disease-like pathology through Akt-ERK crosstalk pathway in male rats

**DOI:** 10.1007/s00213-024-06697-4

**Published:** 2024-10-23

**Authors:** Mhasen Khalifa, Rabie H. Fayed, Yasmine H. Ahmed, Mohamed F. Abdelhameed, Ahmed F. Essa, Heba M. A. Khalil

**Affiliations:** 1 Department of Veterinary Hygiene and Management, Faculty of Veterinary Medicine, Cairo University, Giza, 12211 Egypt; 2 Cytology and Histology Department, Faculty of Vet. Medicine, Cairo University, Giza, 12211 Egypt; 3 Pharmacology Department, National Research Centre, 33 El Bohouth St., Dokki, Giza 12622 Egypt; 4 Department of Natural Compounds Chemistry, National Research Centre, Dokki, Giza 12622 Egypt; 5 Faculty of Veterinary medicine, King Salman International University, South sinai Ras Sudr, Egypt

**Keywords:** BPA, Neurotoxicity, Ferulic acid, Oxidative stress, Apoptosis, Molecular docking, Beta-amyloid 1–42, AChE, Total tau protein, Phosphorylated tau protein

## Abstract

**Objectives:**

This study investigated the neuroprotective effect of ferulic acid (FA) against bisphenol A (BPA) induced Alzheimer’s disease-like pathology in male rats.

**Methods:**

Rats were allocated into four groups, control, BPA, BPA + FA, and FA, respectively, for 40 days. Spatial working memory and recognition memory were evaluated. Moreover, the brain levels of oxidative stress biomarkers, proinflammatory cytokines, extracellular signal-regulated kinase (ERK), and phosphorylated serine/threonine protein kinase (p-Akt) were measured. We also determined the brain neuropathological protein levels, including Beta-Amyloid 1–42, total Tau (tTau), and phosphorylated Tau (pTau) proteins. Furthermore, brain levels of Acetylcholinesterase (AChE) and Beta-secretase (BACE) were assessed. Brain histological investigation and immunohistochemistry determination of glial fibrillar acidic protein (GFAP) were also performed. Moreover, docking simulation was adapted to understand the inhibitory role of FA on AChE, BACE-1, and ERK1/2.

**Results:**

Interestingly, the BPA + FA treated group showed a reversal in the cognitive impairments induced by BPA, which was associated with improved brain redox status. They also exhibited a significant decrease in brain inflammatory cytokines, ERK, and p-Akt levels. Moreover, they revealed a decline in beta-amyloid 1–42 and a significant improvement in tTau expression and pTau protein levels in the brain tissue. Further, the brain levels of AChE and BACE were substantially reduced in BPA + FA rats. The neuroprotective effect of FA was confirmed by restoring the normal architecture of brain tissue, which was associated with decreasing GFAP.

**Conclusion:**

FA could be a potent neuroprotectant agent against AD with a possible prospect for its therapeutic capabilities and nutritional supplement value due to its antioxidant and antiapoptotic properties.

**Supplementary Information:**

The online version contains supplementary material available at 10.1007/s00213-024-06697-4.

## Introduction

Bisphenol A (BPA, 2, 2′ -bis (4-hydroxyphenyl) propane) is an environmentally toxic chemical used in the manufacture of polycarbonate plastic and epoxy resin (Calafat et al. 2008). BPA can be readily dissociated from food and beverage containers, making oral exposure the main route of BPA exposure (Vandenberg et al. 2009; Almeida et al. 2018). According to previous reports, approximately 3.5 million tons of BPA are produced annually (Huo et al. 2015). The U.S. Environmental Protection Agency (EPA) and the U.S. Food and Drug Administration (FDA) established a BPA reference dose of 50 µg/kg B.W./day (Vandenberg et al. 2010; Gore et al. 2015), while the European Food Safety Authority (EFSA) estimated as tolerable a daily intake (TDI) of 4 µg BPA/kg B.W./day (EFSA Panel on Food Contact Materials and Aids (CEF) 2015).

BPA is a xenoestrogen and a well-known Endocrine Disrupting Chemical (EDC) that can bind to estrogenic receptors and interfere with steroid signaling (Russo et al. 2019). Additionally, it can bind to various hormonal receptors, resulting in hormonal disruption, including testosterone hormones (Xu et al. 2005; Tanaka et al. 2006), Thyroid hormones (Moriyama et al. 2002; Chevrier et al. 2013) and glucocorticoids (Poimenova et al. 2010). BPA can cross the blood-brain barrier and accumulate in brain tissue (Vandenberg et al. 2007; Wang et al. 2019). It induced neurobehavioral problems in children, including anxiety and depression, and also affected the spatial and avoidance memory of male mice (Xu et al. 2010; Perera et al. 2016). In addition, BPA exposure causes brain inflammation, resulting in cognitive impairment and neurological diseases like Alzheimer’s and Parkinson’s (Niranjan 2018; Sukjamnong et al. 2020).

BPA disrupts the balance between ROS/RNS production and endogenous antioxidant defense systems, causing brain oxidative stress (Khadrawy et al. 2016; Haridevamuthu et al. 2022). Also, BPA reduces antioxidant enzymes (particularly superoxide dismutase, catalase, GSH reductase, and GSH peroxidase) and raises thiobarbituric acid-reactive substances (Jain et al. 2011). Moreover, oxidative stress could deactivate the structural and functional mechanisms of several enzymes and proteins involved in cell signaling, leading to a decline in synaptic plasticity, neuronal cell death, memory impairment, and cognition dysfunction (Jain et al. 2011; Massaad and Klann 2011; Haddadi et al. 2014).

The extracellular signal-regulated kinase (ERK) subgroup of mitogen-activated protein kinases (MAPK) regulates cell proliferation, survival, differentiation, and apoptosis (Cagnol and Chambard 2010). ERK is activated to initiate cell damage and apoptosis in response to toxic insults and endogenous stimuli in mammalian cells (Lu et al. 2014; Fu et al. 2020). Furthermore, Akt (protein kinase B), a serine/threonine-specific protein kinase family member, is regarded as a crucial regulator in signal pathways, such as cell survival, growth, proliferation, migration, and apoptosis, by phosphorylating a variety of intracellular proteins (Franke et al. 2003; Manning and Cantley 2007). Interference of ERK and Akt-mediated signaling significantly contributed to the pathogenesis of various diseases, including diabetes and neurodegenerative disorders (D’Angelo et al. 2019; Rai et al. 2019). Moreover, activation of the PI3-K/Akt and ERK pathways implicated in neuroprotection and neurotoxicity prevents neuronal apoptosis, which has led to progress in developing a therapeutic molecule targeting Akt and ERK signaling pathways for neurodegenerative diseases (Cui et al. 2011; Rai et al. 2019). According to several studies, BPA can trigger neuronal cytotoxicity and apoptosis via ERK or Akt downstream-regulated pathways, resulting in neuronal cell death (Lee et al. 2008; Wang et al. 2019; Huang et al. 2021).

Alzheimer’s disease-like changes had been reported following BPA exposure in both adults and prenatally. These changes include elevated amounts of pathogenic proteins APP, Aβ, and p-Tau (Fang et al. 2015, 2016). BPA significantly increases oxidative stress and Aβ in the cortex and hippocampus of the AD brain while also raising neuroinflammation and tau-phosphorylation (Abdel-Rafei and Thabet 2020). BPA also decreased hippocampal spine density and disrupted the equilibrium between kinase and protein phosphatase, which regulate p-Tau in animals exposed to BPA during pregnancy (Kimura et al. 2016; Xue et al. 2020).

Ferulic acid (4-hydroxy-3-methoxycinnamic acid, FA) is a cinnamic acid derivative. FA is a member of the hydroxycinnamic acid family derived from the leaves and seeds of *Ferula asafoetida* (Sosulski et al. 1982; Lempereur et al. 1997). FA is a natural antioxidant compound that widely exists in foods and herbal medicines (Neto-Neves et al. 2021). FA has been reported to have strong antioxidant activity, anti-inflammatory, anticancer, and antifibrosis effects (Rice-Evans et al. 1996; Yan et al. 2013; Gao et al. 2018; Li et al. 2021; Ali et al. 2021). As well as FA has revealed a potent cytoprotective action by scavenging free radicals (Zhang et al. 2019). Also, FA can cross the blood-brain barrier and induce anti-depression and prokinetics in the hippocampus of male rats (Zhang et al. 2011). The level of FA was sufficient to trigger antioxidant capabilities and decrease intracellular reactive oxygen species (ROS) as evaluated by 2′,7′-dichlorofluorescein diacetate (DCFDA) in an Alzheimer’s disease model (Zafeer et al. 2019). FA showed several ameliorative effects on neurobehavioral impairments, such as depressive-like behavior, Aβ-induced memory loss, and cognitive deficiencies, as well as a neuroprotective impact against ischemia/reperfusion-induced brain injury (Sgarbossa et al. 2015; Kikugawa et al. 2016; Hassanzadeh et al. 2017; Ren et al. 2017; Singh et al. 2017).

FA has been shown to mitigate AD-like pathology by inhibiting hippocampus capillary density and diameter loss, which is crucial for Aβ plaque deposition and spatial memory deficit in animal models (Wang et al. 2021; Park et al. 2022). FA also reduces AD-like pathological alterations in the hippocampus, such as Aβ deposition and Tau phosphorylation, which may be connected with the suppression of inflammatory cytokines (Jung et al. 2016; Wang et al. 2017a; Saini et al. 2021). Furthermore, FA preserved the architecture of pyramidal cells in the hippocampus of an Alzheimer’s disease-induced model, indicating substantial antioxidant activity (Zafeer et al. 2019). In addition, it has been verified to be highly effective in treating Alzheimer’s disease and memory impairment caused by Aβ deposition (Sgarbossa et al. 2015; Kikugawa et al. 2016).

This study attempts to examine the neuroprotective effect of ferulic acid on BPA-induced AD-like pathology by evaluating spatial working and recognition behavior as well as brain oxidative stress, proinflammatory cytokines, and the neuropathological hallmark of AD, including beta-amyloid (1–42) and total and phosphorylated Tau protein deposition levels. Moreover, we carried out molecular studies for AChE, BACE-1, and ERK1/2, supported by in vitro assessment of their concentrations in the brain.

## Materials and methods

### Chemicals

Bisphenol A and FA powder were purchased from Sigma Aldrich (St Louis, Missouri, USA). The ReadyPrepTM protein extraction kit (total protein) provided by Bio-Rad Inc (Catalog #163–2086) was employed according to manufacturer instructions and was added to each tissue homogenate of all different groups. Bradford Protein Assay Kit (SK3041) for quantitative protein analysis was provided by Bio basic Inc (Markham Ontario L3R 8T4 Canada).

### Experimental design

#### Animals

*Wistar* rats, weighing around 120 ± 30 g and with an average age of 7–8 weeks, were acquired from VACCERA, Egypt. They were adapted for one week in a shoebox plastic cage under controlled conditions consisting of a 12-hour light/dark cycle, 50 ± 10% humidity, and 25 ± 3 °C temperature. All animals were allowed free access to food and water. All rats were utilized following VET-IACUC regulations (Vet CU 03162023744).

#### Treatment groups

**Fig. 1:**
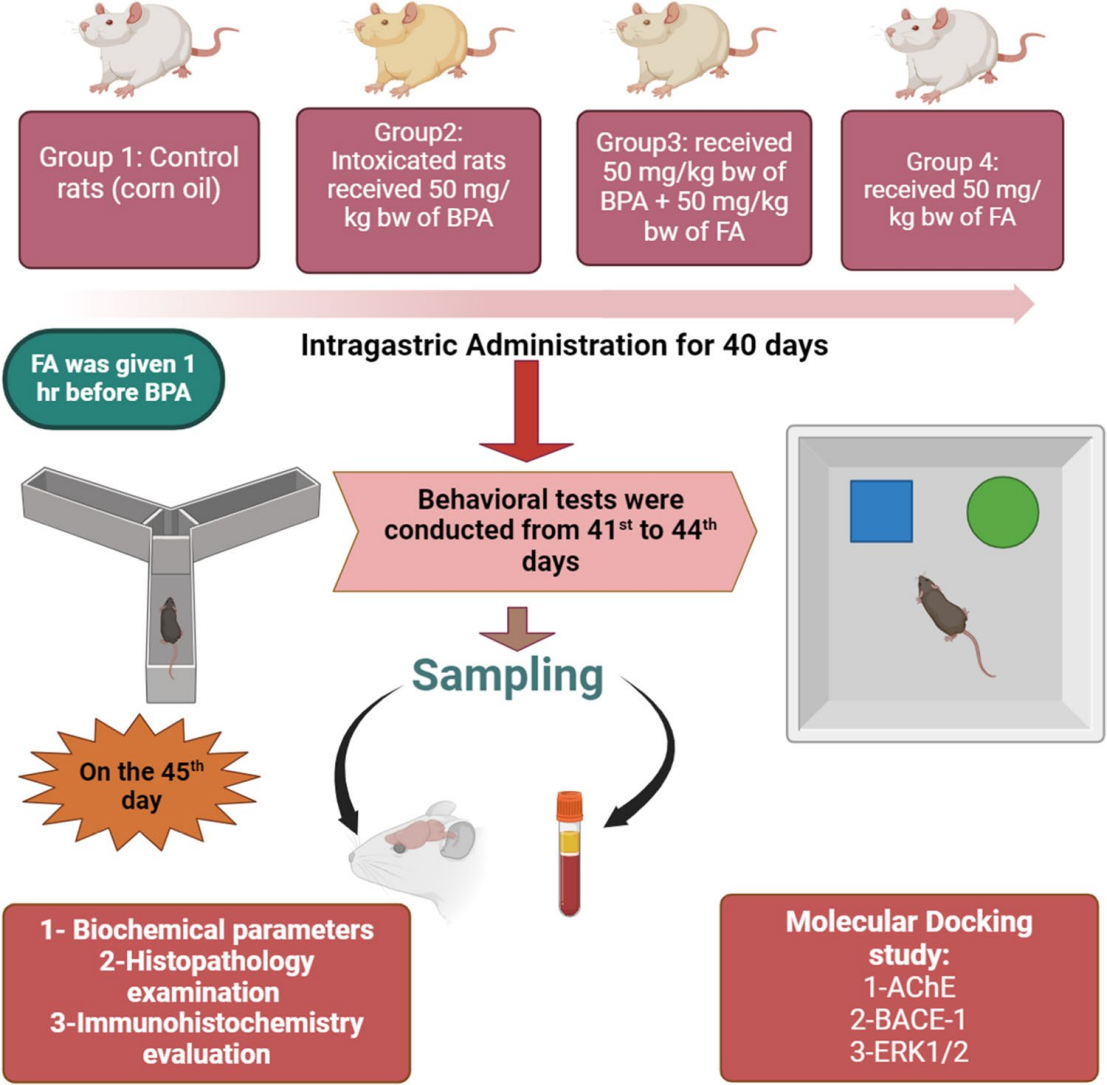
Diagram illustrating the experimental procedure. Male rats were allocated to four groups: control, BPA-intoxicated rats, rats co-treated with BPA + FA, and FA group. All agents were given 40 days; after that, behavioral tests, including Y-maze and NOR, were evaluated from the 41st to the 44th. On the 45th day, blood and brain samples were obtained to evaluate changes in biochemical, histological, and immunohistochemistry parameters

Forty male rats were divided into four groups of 10 animals each. Group 1 control rats received 2 ml /kg corn oil (vehicle), group 2 intoxicated rats received 50 mg/kg bw of BPA dissolved in corn oil, and group 3 treated group received 50 mg/kg bw BPA + 50 mg/kg bw of FA which were dissolved in corn oil. While group 4 received 50 mg/kg bw of FA dissolved in corn oil. All the corresponding drugs were administrated via oral gavage for 40 days. FA was given one hour before the BPA in group 3 rats, as shown in (Fig. 1).

### Dose selection rationale

The U.S. EPA defines “low-dose” impacts of environmental EDC as effects reported for chemicals at doses lower than those utilized in conventional toxicologic research conducted for risk assessment purposes (vom Saal and Hughes 2005). Additionally, “low dose” is frequently used to describe environmentally relevant doses, i.e., doses that produce serum levels comparable to those found in human serum (Richter et al. 2007). As for BPA, before 1997, the lowest dose studied for risk assessment was 50 mg/ (kg per day), which is considered the accepted lowest observed adverse effect level (LOAEL) currently in the USA, that was used to calculate the current EPA reference dose (and FDA-acceptable daily intake or ADI dose) of 50 µg/ (kg day); this presumed “safe” dose is estimated by dividing the LOAEL by three 10-fold safety factors (i.e., by 1000) (Vandenberg et al. 2013). Meanwhile, the dose of FA was selected based on previous reports (Yu et al. 2021; Park et al. 2022).

### Behavioral assessments

All behavioral tests were conducted 24 h after the last dose of treatment, from the 41st day to the 44th day between 9:00 a.m. and 3:00 p.m., as illustrated in (Fig. 1). All behavioral devices were touched with 70% alcohol between rats to eliminate any olfactory cues that could bias our results.

#### Y- maze test

Short-term working memory was evaluated using the Y-maze test (Kokkinidis et al. 1976; Kraeuter et al. 2019). This test is based on the rodents’ natural tendency to explore a new environment without negative or positive reinforcement and under minimal stress (Sarnyai et al. 2000). Y-maze apparatus consisted of three wooden arms (A, B, and C) with a length of 40 cm, width of 10 cm, and height of 35 cm, joined at an angle of 120°. In brief, each rat is put at the end of one arm and allowed to explore the maze for 5 min (Khalil et al. 2021). The number of arm entries and spontaneous alternations percentage (SAP) were recorded. The SAP was calculated using the following equation= (spontaneous alternation/number of arm entry-2 × 100) (Shcherbakova et al. 2023).

#### Novel object recognition (NOR) test

NOR test was used to evaluate the hippocampus-dependent memory impairment (Lueptow 2017). This test is based on the willingness of rats to explore novel objects rather than familiar objects (Ennaceur and Delacour 1988). Three consecutive days were devoted to a novel object recognition test comprising three phases. The first day was the habituation phase, in which rats were put in a wooden apparatus (70 × 70 × 35 cm) and allowed to explore the arena for 5 min. Then, on the second day, there was an acquisition phase in which rats were placed with two identical objects for 5 min. The last phase was the recall phase, in which rats were placed in the arena with one familiar object and one novel object and allowed to explore the two distinct objects for 5 min (Khalil et al. 2020). The preference for novel object % and the discrimination ratio were calculated as follows =

$$\begin{array}{l}\mathrm{The}\;\mathrm{preference}\;\mathrm{for}\;\mathrm{novel}\;\mathrm{object}\;\%=\mathrm{Novel}\;\mathrm{object}\;\mathrm{exploration}\;\mathrm{time}\;/\;\mathrm{Total}\;\mathrm{exploration}\;\mathrm{time}\;\times100\\\mathrm{DR}\;=\;\left(\mathrm{Novel}\;\mathrm{object}\;\mathrm{exploration}\;\mathrm{time}-\mathrm{old}\;\mathrm{object}\;\mathrm{exploration}\;\mathrm{time}\right)\;/\;\left(\mathrm{Novel}\;\mathrm{object}\;\mathrm{exploration}\;\mathrm{time}\:+\:\mathrm{old}\;\mathrm{object}\;\mathrm{exploration}\;\mathrm{time}\right).\end{array}\\$$

### Blood collection and tissue preparation

After 24 h of the last behavioral test, blood samples were obtained from the rats’ retro-orbital venous plexus and allowed to coagulate. Then, samples were centrifuged at 3000 rpm for 10 min. The collected sera were stored at − 20 °C to determine the oxidative stress parameters. After collecting blood samples, rats were sacrificed by cervical dislocation. Their brains were gently removed and immediately rinsed with ice, then homogenized in ice-cold phosphate buffer saline (20% w.v). The homogenate was centrifuged at 10,000 rpm for 20 min to remove the cell debris, unbroken cells, nuclei, erythrocytes, and mitochondria. The supernatant (cytoplasmic extract) was used to estimate beta-amyloid 1–42, ERK, TNF-α, IL-1β, p-Akt, AChE, BACE, and pTau according to manual instructions. Also, halves of these brains were fixed for 48 h in 10% neutral buffered formalin for histological and immunohistochemical analysis.

### Biochemical parameters

The lipid peroxidation marker (MDA) and oxidized glutathione (GSSG) were assayed calorimetrically by a kit from Bio Vision Incorporated, San Francisco, USA). Meanwhile, reduced glutathione (GSH) was determined calorimetrically using Biodiagnostic kits (Cairo, Egypt). In the brain homogenate, the Rat Beta Amyloid 1–42, ERK, TNF-α, IL-1β, and p-Akt were determined by ELISA kit (Sandwich ELISA, Lifespan BioSciences, Seattle, USA). In addition, the AChE was determined using an ELISA kit supplied by CUSABIO Technology LLC, China (Cat. # CSB-E11304r). While the β-Secretase Fluorometric Assay Kit (Cat #K360-100) was purchased from Biovision, CA, USA for measuring BACE as well as pTau measured by using Rat Tau ELISA Kit (Cat # NBP2–81164, Novus Biologicals, LLC, USA). All experiments were performed according to the manufacturer’s instructions.

### Western blot

After brain tissue samples were collected from each animal, the tissue homogenization was performed in an ice-cold RIPA buffer (Thermofisher Scientific, Waltham, MA, USA) supplemented with a protease and phosphatase inhibitor cocktail (Sigma-Aldrich, St. Louis, MO, USA) for 30 min. Supernatants containing the total protein fraction were collected after centrifugation at 14,000× g for 15 min.

To determine protein concentration in each sample. A Bradford assay was carried out according to manufacturer instructions and as described by a previous study (El-Hawary et al. 2021). Briefly, an equal amount of protein concentration from each sample (20 µg) was loaded with an equal volume of (2x Laemmli sample buffer containing 4% SDS, 10% 2-mercaptoethanol, 20% glycerol, 0.004% bromophenol blue and 0.125 M Tris HCl) in optimum PH situation (6.8). Before loading on polyacrylamide gel electrophoresis, denaturation was done by boiling each previous mixture at 95 °C for 5 min. Then, the separation by electrophoresis was performed using TGX Stain-Free™ FastCast™ Acrylamide Kit (SDS-PAGE), which was provided by Bio-Rad Laboratories Inc Cat # 161–0181, and then transferred to polyvinylidene fluoride (PVDF) membranes. These Membranes were blocked with Tween 20 (TBST) buffer and Bovine Serum Albumin (BSA)3% at room temperature for one hr. to avoid non-specific binding.

The primary antibody of Tau was purchased and then diluted in TBST according to manufactured instructions. Incubation of membranes with primary antibody solution, either TAU or b-actin, was done overnight at 4 °C. The washing process was done in two steps by rinsing with TBST 3–5 times for 5 min, between them the Incubation in the HRP-conjugated secondary antibody (Goat anti-rabbit IgG- HRP-1 mg Goat mab -Novus Biologicals) solution against the blotted target protein for 1 h. at room temperature happened. Finally, images were captured after adding the chemiluminescent substrate (Clarity TM Western ECL substrate Bio-Rad cat#170–5060) using a CCD camera-based imager, and bands were analyzed using ImageJ software 1.53t (National Institutes of Health, Bethesda, Maryland, USA) against control sample beta-actin (housekeeping protein). All fold changes of band densities were determined with normalization to β-actin, an endogenous control. Relative protein expression was calculated as the relative density of a protein band normalized to the endogenous control. Each experiment was conducted in triplicate and repeated three times independently.

### Histological examination

The preserved brain tissues were dehydrated in ascending degrees of ethyl alcohol, cleared in xylene, and embedded in paraffin wax. Rotatory microtome sections of 4 μm thickness were produced, deparaffinized, and stained with hematoxylin and eosin (H&E) stain for light microscopic examination (Bancroft and Gamble 2013) and immunohistochemistry.

### Immunohistochemical examination of glial fibrillar acidic protein (GFAP)

GFAP was used to detect astrocyte protein according to Stoltenburg-Didinger et al. (1996). Sections stained with anti-GFAP were analyzed using a digital Leica Quin 500Â image analysis system (Leica Microsystems, Switzerland) housed at the Faculty of Dentistry, Cairo University.

### 10. Molecular docking studies (experimental)

For carrying out the docking process, Molecular Operating Environment (MOE) software version 2015.10 was used. Initially, the catalytic domains of Recombinant Human Acetylcholinesterase (AChE) (PDB ID: 4EY6) (Cheung et al. 2012), Beta-Secretase 1 (BACE-1) (PDB ID: 7N66) (Rombouts et al. 2021), and the extracellular signal-regulated kinase 1/2 (ERK1/2) (PDB ID: 6G97) (Heightman et al. 2018) were extracted from the Protein Data Bank. The proteins were prepared by eliminating unwanted solvents and other cofactors and using the default MOE “QuickPrep” module parameters. The potential binding pockets encompassing the critical residues were then prepared using the ‘Site Finder’ feature. For the validation step, the ligands were re-docked in their binding site, showing most of the critical interactions at an acceptable RMSD. The energy-minimized FA database file (.mdb) was created and docked in the binding site. The results were displayed as ΔG ^*a*^ (kcal/mol) with RMSD values of 2 (Table 1), and the best conformers’ 2D and 3D representations were examined (Figs. 9, 10, and 11).

**Table 1:** Results of docking simulations of the ferulic acid

No.	Name	ΔG ^a^ (kcal/mol)		
		AChE	BACE-1	ERK1/2
1	Ferulic acid	–5.39	–4.60	–5.39
2	The co-crystallized ligand	–7.05	–9.19	–8.57

### Statistical analysis

All behavioral, biochemical, and histopathological parameter results were expressed as mean ± standard error of the mean (SEM). Statistical analysis was performed using GraphPad Prism software (Version 7.00). All evaluations using one-way ANOVA were followed by the Tukey test to determine statistical differences among the experimental groups. The difference was considered significant at *p* < 0.05.

## Results

### Effect of FA on the cognition deficits induced by BPA

**Fig. 2:**
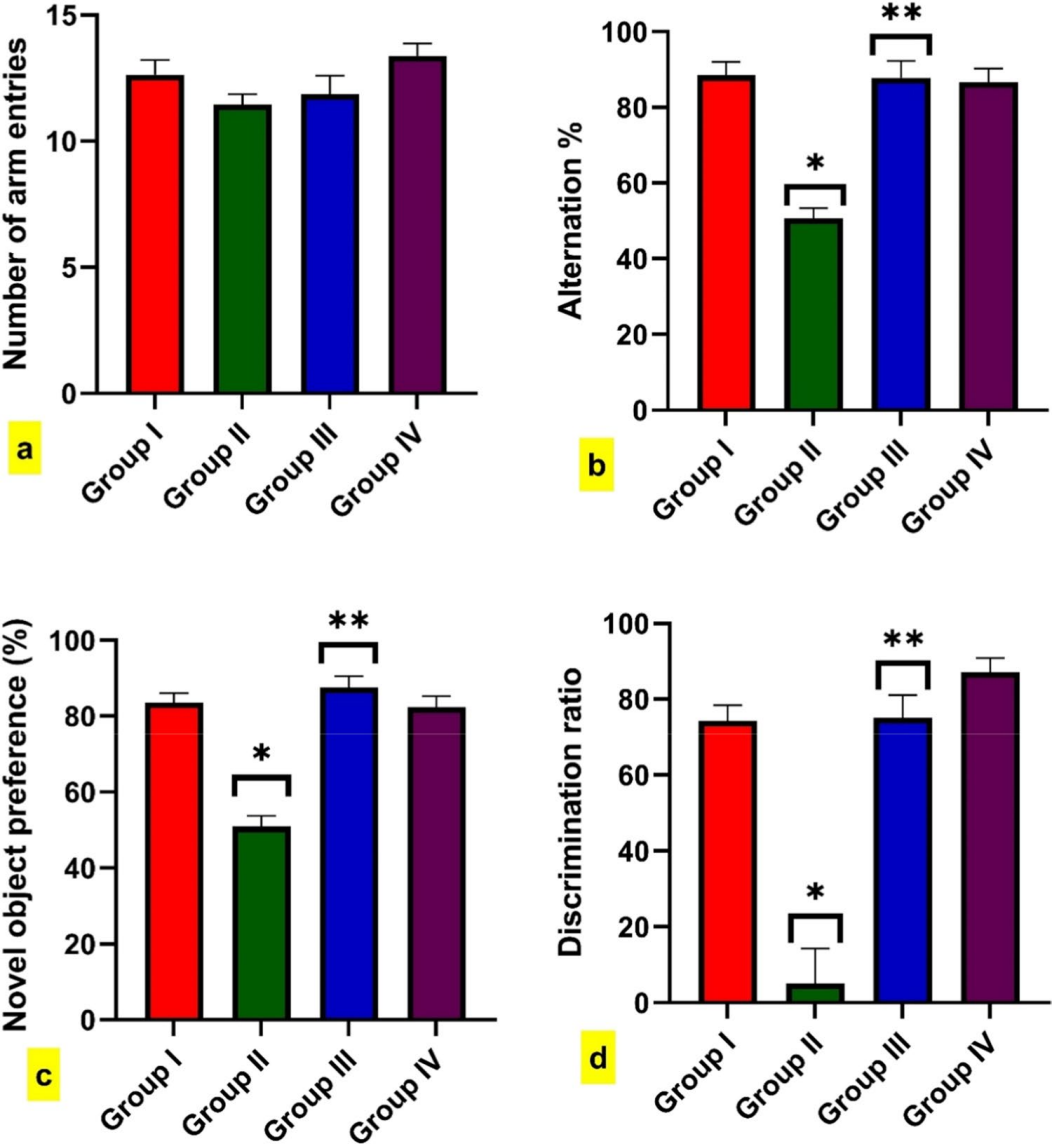
Effect of BPA administration for 40 days on male rats’ spatial working memory and recognition memory. Y-maze test: number of arm entries, **b**. Y-maze test: spontaneous alternation percentage, **c**. Novel object recognition test: novel object preference %, and **d**. Novel object recognition test: Discrimination ratio. Data are expressed as mean standard error of the mean (SEM) (one-way analysis of variance (one-way ANOVA) followed by Tuckey post hoc test for ten rats in each group. * Significantly different from Group I (control group) and ** significantly different from Group II (BPA-intoxicated rats),*P* < 0.05

As depicted in (Fig. 2a), there are no significant differences between groups regarding the number of arms entries. Conversely, BPA-intoxicated rats exhibited a marked reduction in SAP % compared to the control group. Also, intoxicated rats treated with FA demonstrated a significant increment of alternation % compared to BPA-intoxicated rats (Fig. 2b). In NOR, BPA-intoxicated rats showed a substantial decrease in novel object preference percentage and discrimination ratio compared to control rats. Moreover, BPA + FA treated rats displayed a significant increase in the proportion of rats that preferred novel objects and the discrimination ratio compared to BPA-intoxicated rats (Fig. 2c, d).

### Effect of FA on the level of brain oxidative stress biomarkers in BPA-intoxicated rats

**Fig. 3:**
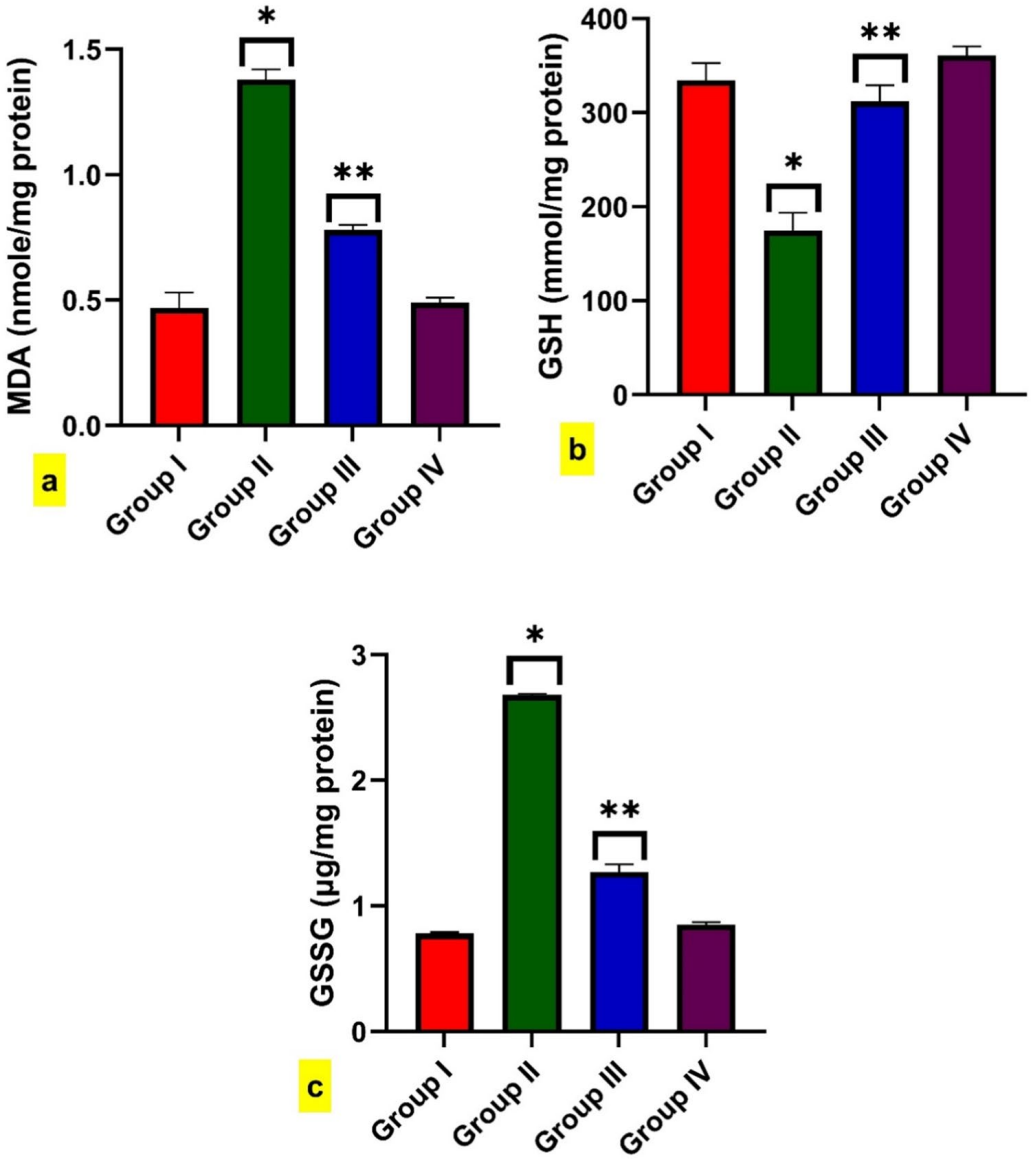
Effect of BPA administration for 40 days on the level of oxidative stress markers in the brain, such as (**a**) MDA, **b** GSH, and (**c**) GSSG. Data are expressed as mean ± standard error of the mean (SEM) (one-way analysis of variance (one-way ANOVA) followed by Tuckey post hoc test for ten rats in each group. * Significantly different from Group I (control group) and ** significantly different from Group II (BPA-intoxicated rats), *P* < 0.05

BPA-intoxicated rats exhibited a significant increment in MDA compared to control rats. While rats treated with BPA + FA revealed a substantial reduction in MDA levels compared to rats intoxicated with BPA (Fig. 3a). Moreover, males intoxicated with BPA displayed a considerable decline in GSH and a significant rise in GSSG compared with the control. In contrast to the toxicity group (Group II), rats co-treated with BPA + FA revealed a significant increment of GSH associated with a significant decrease in GSSG (Fig. 3b, c). However, rats treated with FA did not differ significantly from control rats.

### Effect of FA on the amyloid 1–42 protein level in BPA-intoxicated male rats

**Fig. 4:**
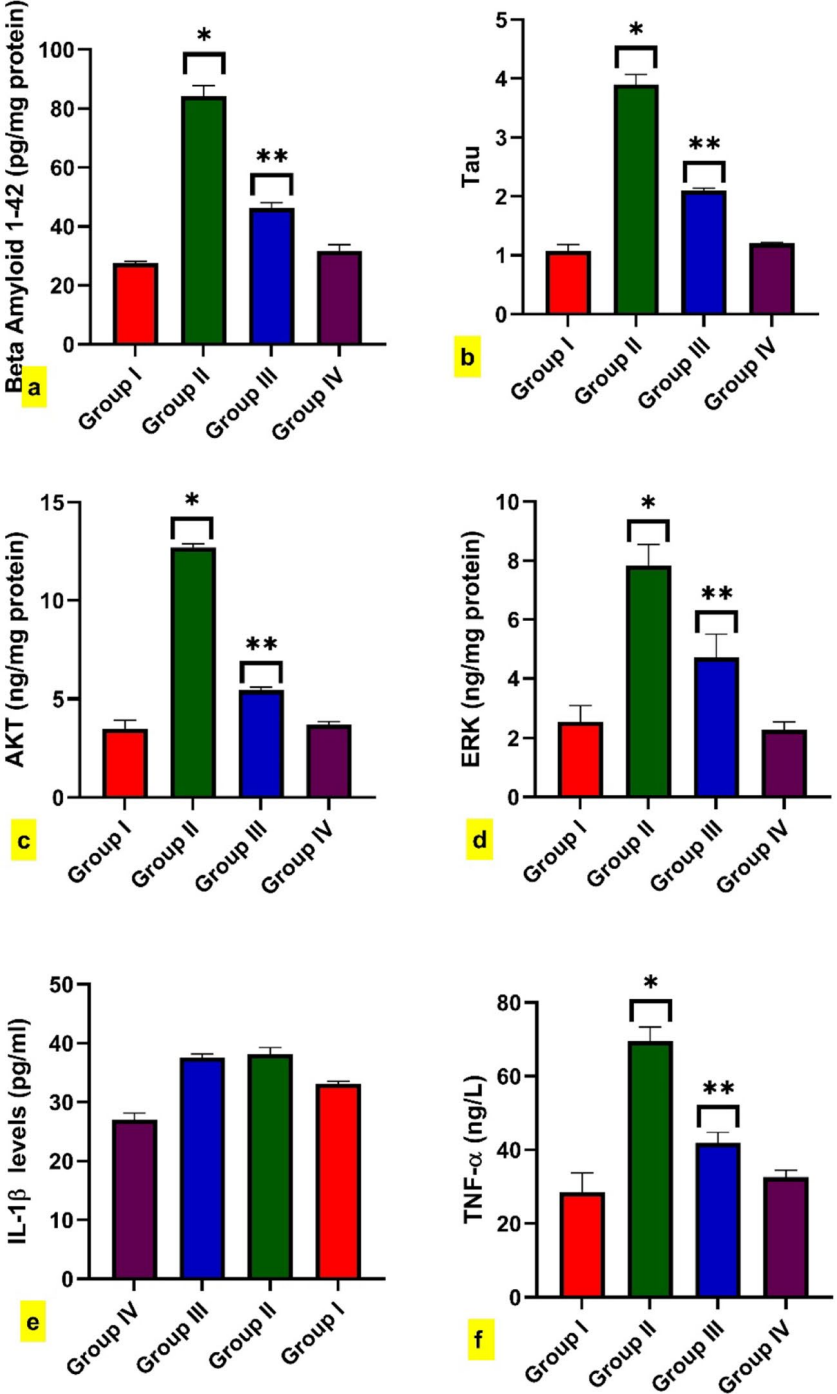
Effect of BPA administration for 40 dayson the brain level of (**a**). Beta-amyloid, **b**. tTau. Data, **c**. p-Akt, **d**. ERK, as well as pro-inflammatory cytokines, **e**. Interleukin 1 beta, and **f**. Tumor necrosis factor-alpha. Data are expressed as mean ± standard error of the mean (SEM) (one-way analysis of variance (one-way ANOVA) followed by Tuckey post hoc test for ten rats in each group. * Significantly different from Group I (control group) and ** significantly different from Group II (BPA- intoxicated rats), *P* < 0.05

The amyloid 1–42 protein level exponentially increases in rats intoxicated with BPA compared to control rats. Furthermore, rats co-administered BPA + FA revealed a substantial decline in amyloid 1–42 protein levels compared to rats intoxicated with BPA (Fig. 4a).

### Effect of FA on BPA-induced brain inflammation and apoptosis

As shown in (Fig. 4f, c, d), the brain levels of TNF-α, p-Akt, and ERK were significantly elevated in BPA-intoxicated rats in comparison to the control group. While rats co-administrated, BPA + FA revealed a significant reduction in the TNF-α, p-Akt, and ERK compared to toxicity rats (Group II). However, there was no significant elevation between groups in brain levels of IL-1β (Fig. 4e). Also, FA-treated rats did not differ significantly from control rats.

### Effect of FA on the total Tau protein (tTau) expression and phosphorylated Tau (pTau) brain level

**Fig. 5:**
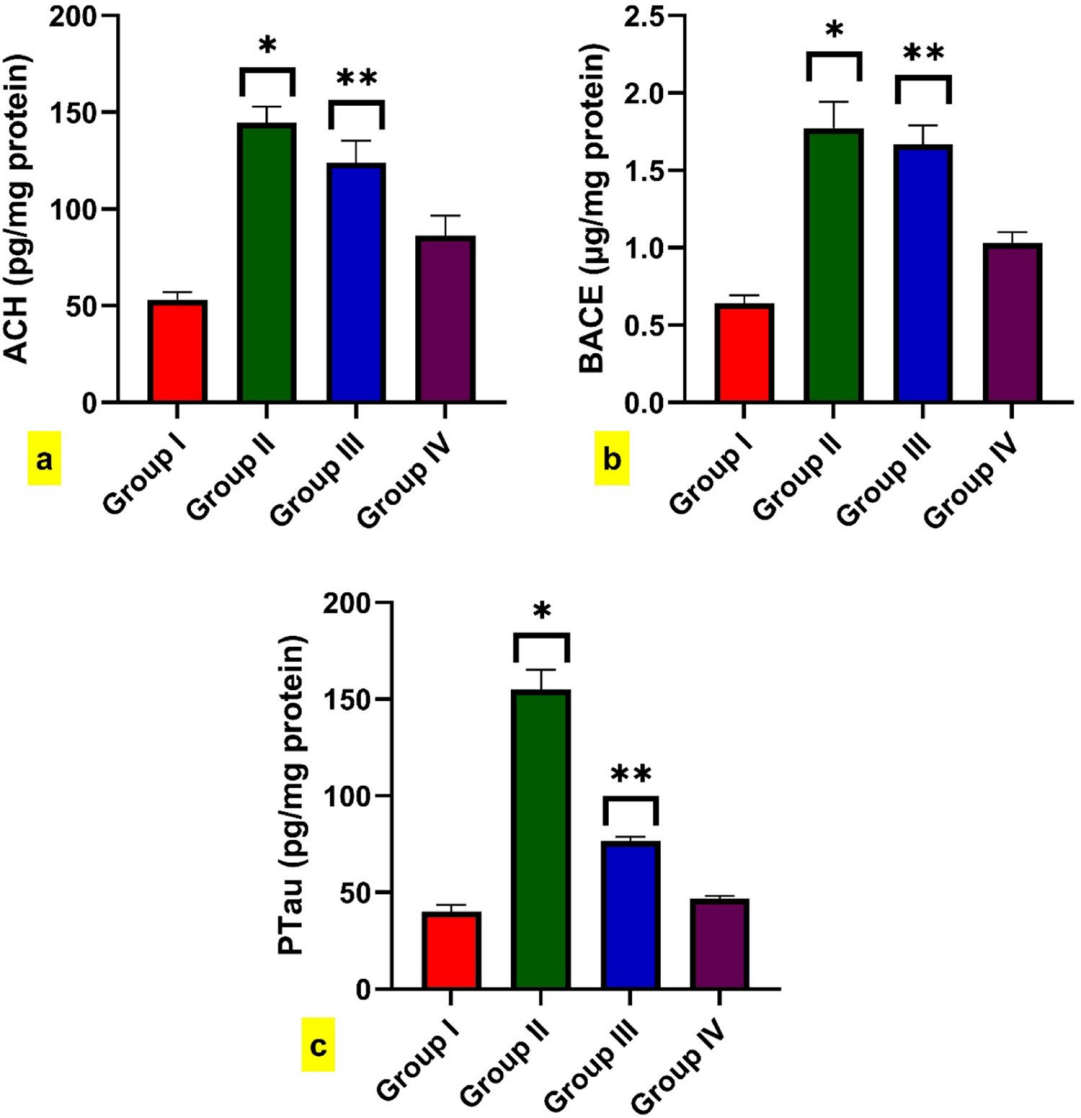
Effect of BPA administration for 40 dayson the brain level of (**a**). AChE, **b**. BACE, and **c**. pTau. Data are expressed as mean ± standard error of the mean (SEM) (one-way analysis of variance (one-way ANOVA) followed by Tuckey post hoc test for ten rats in each group. * Significantly different from Group I (control group) and ** significantly different from Group II (BPA- intoxicated rats), *P* < 0.05

Western blot analysis displayed a higher expression of tTau in BPA-intoxicated rats than in the control group. The administration of FA concurrent with BPA significantly reduced tTau protein expression, as displayed in (Fig. 4b) and the Supplementary file. Moreover, the brain level of pTau exhibited significant rises in BPA-intoxicated rats when contrasted with control rats. However, rats co-administrated with BPA + FA showed a significant decline in the brain level of pTau compared to intoxicated rats. While the FA-treated group did not demonstrate any significant difference from control rats in tTau protein expression and pTau level, as shown in (Fig. 5c).

### Effect of FA on the AChE and BACE brain levels

As presented in **(**Fig. 5a and b), both AChE and BACE levels were significantly increased in the BPA-intoxicated rats compared to the control group. However, the rats co-treated with BPA + FA demonstrated a substantial diminishment in the AChE and BACE brain levels in comparison to rats intoxicated with BPA.

## Histological examination

**Fig. 6:**
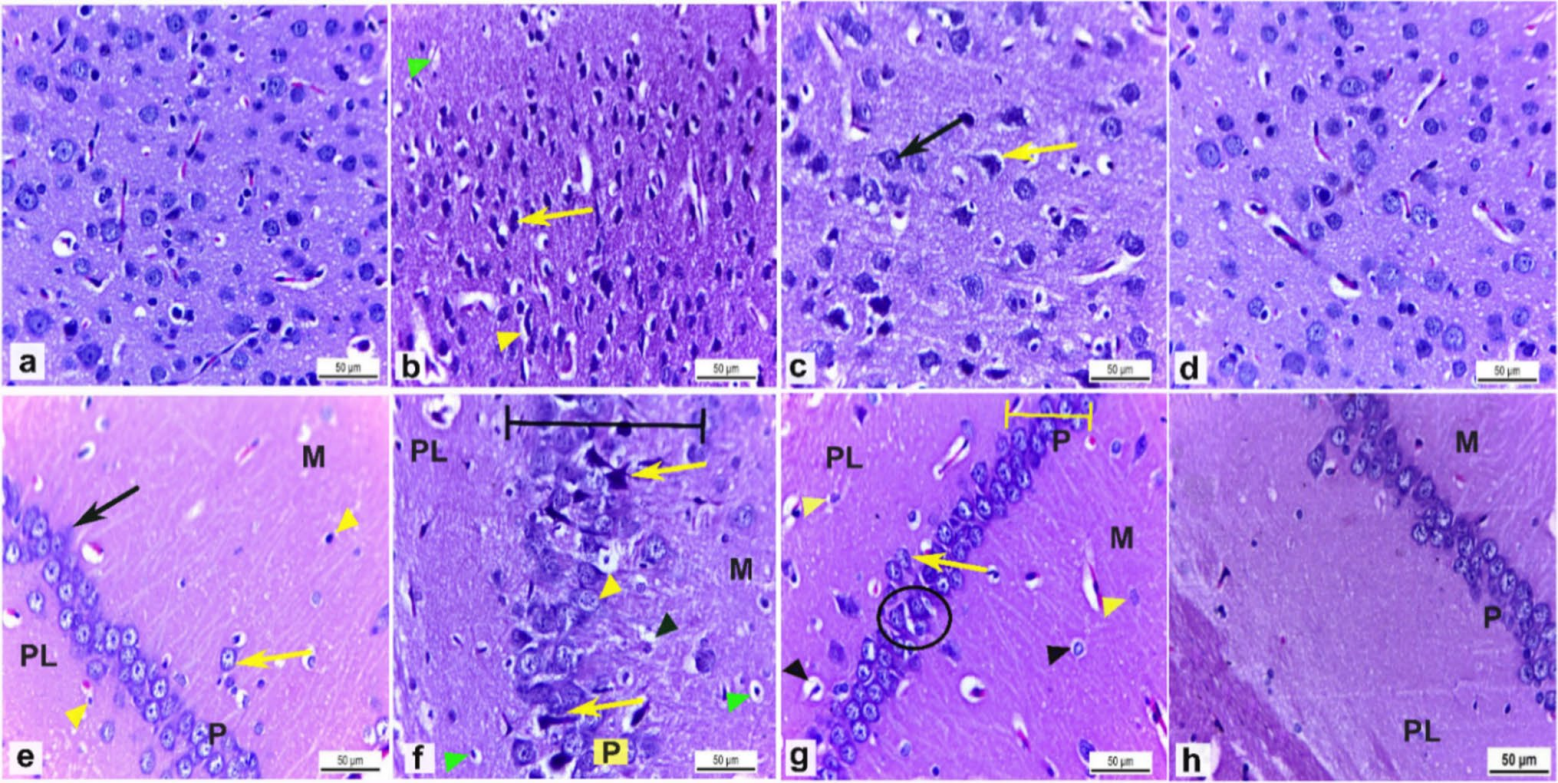
**a**:** h** Brain tissue of male adult Wistar rats. H&E.X400. The cerebral cortex of (**a**) Control rats (Group I) and (**d**) ferulic acid-administered rats (Group IV) showed normal structure and distribution of neurons, neuroglia, and neuropil. **b** Cerebral cortex of Bisphenol A (BPA) administered rats (Group II) showed pyknotic degenerated neurons (yellow arrow) with pericellular spaces (yellow arrowhead) and neuropil vacuolation (green arrowhead). **c** The cerebral cortex of BPA-administered rats plus ferulic acid (Group III) revealed marked recovery in the form of maintenance of the normal architecture of some pyramidal neurons (black arrow), except a few neurons appeared pyknotic with pericellular spaces (yellow arrow). The hippocampus of (**e**) Control rats (Group I) and (**h**) ferulic acid-administered rats (Group IV) showed the normal structure of molecular (M), pyramidal (P), and polymorphic (PL) cell layers, respectively. Molecular (M) and polymorphic (PL) cell layers are composed of scattered neurons (yellow arrow) and neuroglia (yellow arrowhead). The pyramidal cell layer (P) is formed of linearly arranged triangular-shaped neurons with vesicular nuclei (black arrow). **f** Hippocampus of Bisphenol A (BPA) administered rats (Group II) exhibited pyknotic neuroglia with perineural space (green arrowheads) in both molecular (M) and polymorphic (PL) layers. The pyramidal cell layer had disarranged pyramidal neurons (black line), some pyramidal cells appeared pyknotic with pericellular spaces (yellow arrows), and other neurons had nuclei with chromatolysis (yellow arrowhead). There was neuropil vacuolation (black arrowhead). **g** Hippocampus of BPA-administered rats plus ferulic acid (Group III) showed marked maintenance of nearly normal architecture of the three layers. Molecular (M) and polymorphic (PL) layers had normal glial cells (yellow arrowheads), except a few glial cells appeared pyknotic with perineural space (black arrowheads). The pyramidal cell layer (P) revealed normal linear arranged (yellow line) triangular-shaped neurons with vesicular nuclei (yellow arrow), but few neurons appeared irregular in shape with darkly stained cytoplasm and pericellular space (circle)

The cerebral cortex sections of control and FA-administered rats, stained with H&E, showed normal structure and distribution of neurons and neuroglia on the neuropil background (Fig. 6a & d). Contrariwise, the cerebral cortex sections of BPA-intoxicated rats showed pyknotic and degenerated neurons with pericellular spaces and neuropil vacuolation (Fig. 6b). However, cerebral cortex sections obtained from rats administered BPA + FA showed marked recovery compared to the BPA-intoxicated rats in the form of maintenance of the normal architecture of some pyramidal neurons with vesicular nuclei with clear nucleoli. Still, some neurons appeared pyknotic with pericellular space. The neuropil vacuolation diminished (Fig. 6c).

Hippocampus sections of control rats and FA-administered rats showed normal histological architecture of its three layers: molecular, pyramidal, and polymorphic layers, respectively. Molecular and polymorphic layers comprise scattered neurons and neuroglia on the neuropil. The pyramidal cell layer is formed of triangular-shaped neurons arranged in a linear row with vesicular nuclei and prominent nucleoli (Fig. 6e & h). On the other hand, the hippocampus of rats intoxicated with BPA showed pyknotic neuroglia with perineural space in both molecular and polymorphic layers. The pyramidal cell layer exhibited disarrangement of pyramidal neurons; some neurons appeared pyknotic and degenerated with pericellular spaces, and some neurons had nuclei with chromatolysis. There was neuropil vacuolation (Fig. 6f). Meanwhile, the hippocampus of rats administered BPA + FA showed marked maintenance of nearly normal architecture of the three layers compared to the BPA group. Both molecular and polymorphic layers had normal glial cells, except a few glial cells appeared pyknotic with perineural space. The pyramidal cell layer revealed a normal linear arrangement of neurons that appeared nearly normal triangular shaped with vesicular nuclei, but few neurons appeared with an irregular shape, darkly stained cytoplasm, and pericellular space. The neuropil vacuolation diminished (Fig. 6g).

### Immunohistochemical examination

**Fig. 7:**
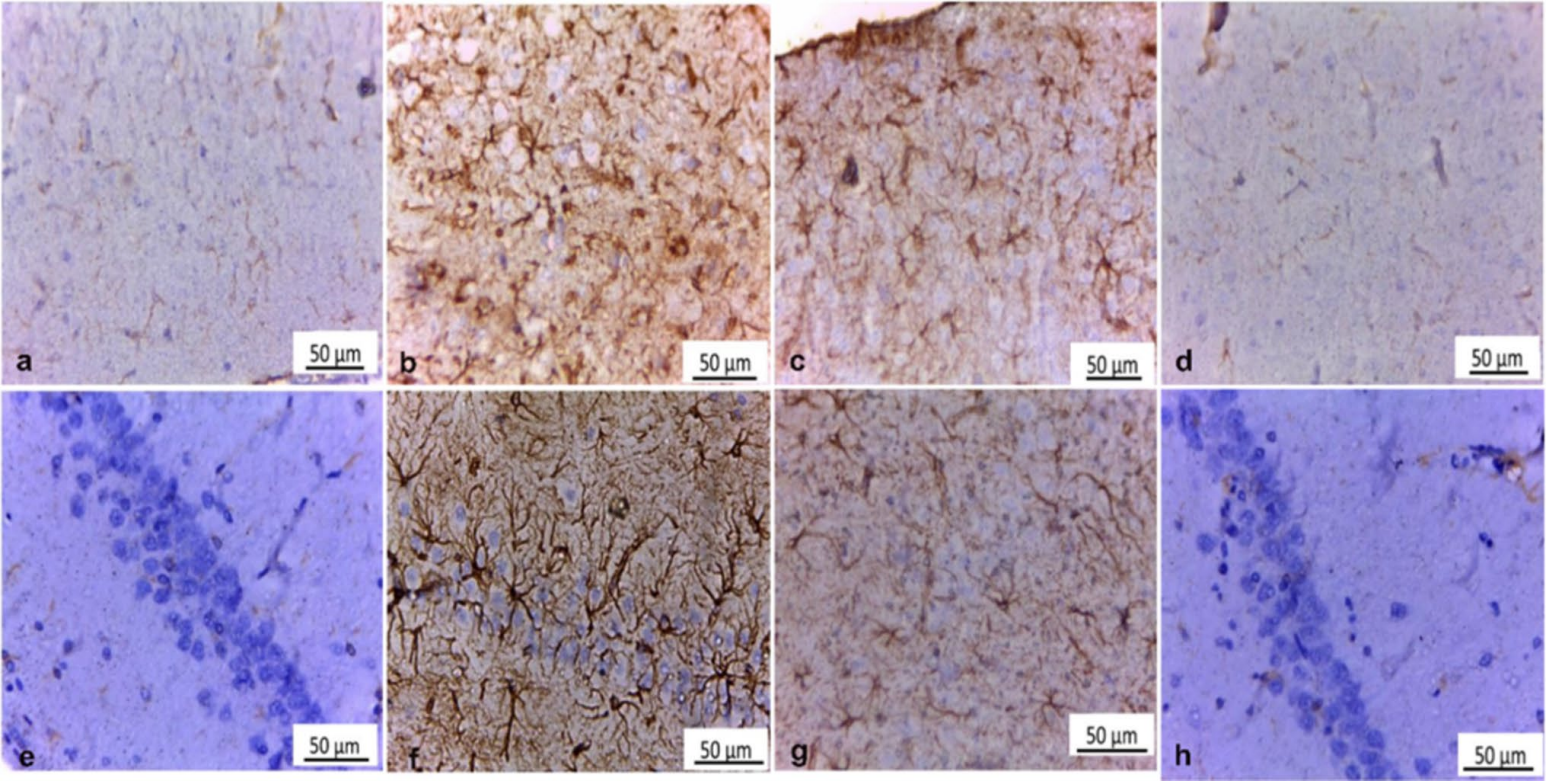
Immunohistochemically GFAP stained cerebral cortex and hippocampus sections (X400). Cerebral cortex and hippocampus (**a **&** e**) of Control rats (Group I) and (**d **&** h**) ferulic acid-administered rats (Group IV) showed weak positive glial fibrillar acidic protein (GFAP) immunoexpression in astrocyte body and processes. **b** Cerebral cortex and (**f**) hippocampus of BPA-administered rats (Group II) revealed strong positive immunoreaction in astrocyte body and processes compared to control rats. **c** Cerebral cortex and (**g**) hippocampus of BPA-administered rats plus ferulic acid (Group III) showed moderate GFAP immunoreactivity in astrocyte body and processes versus the BPA group

**Fig. 8:**
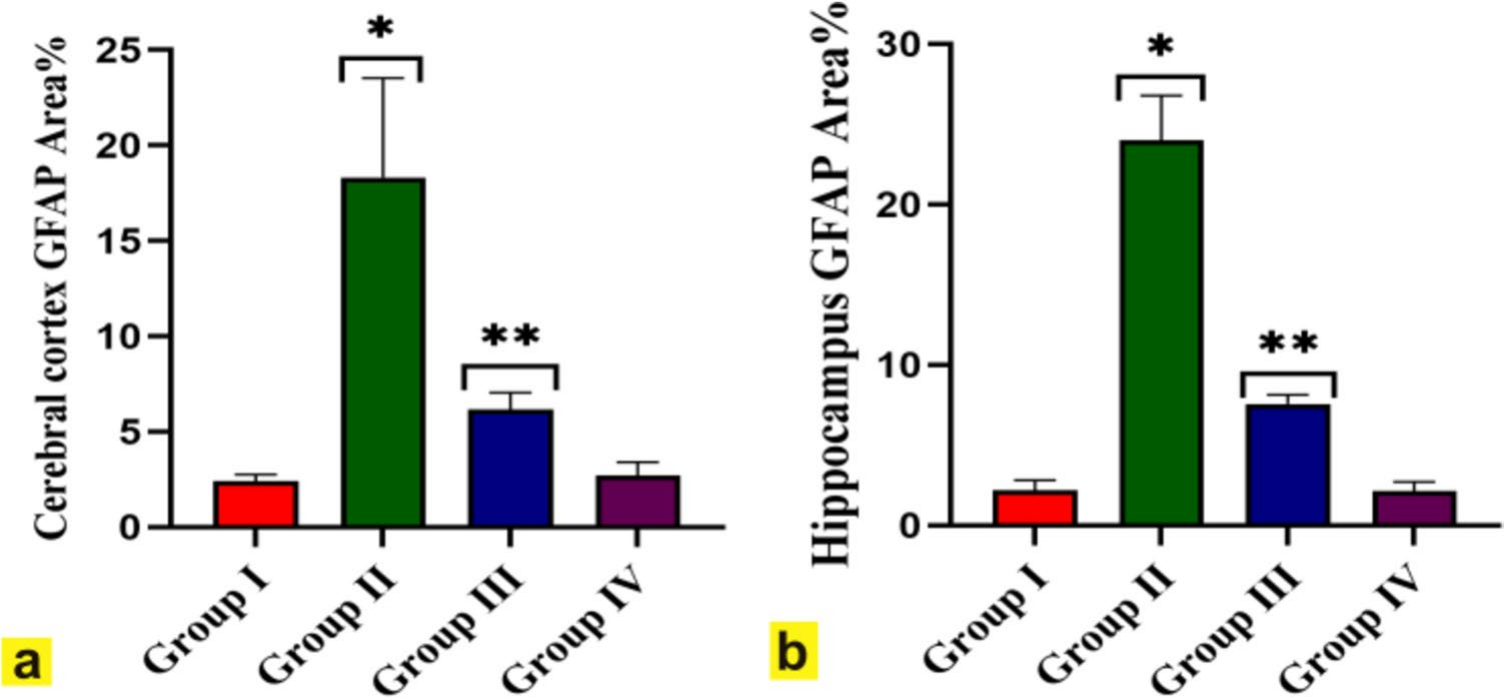
The effect of Bisphenol A (BPA), BPA plus ferulic acid, ferulic acid on the percent area covered by GFAP-positive immunoreactive cells within brain tissue of rats. Results are presented as mean ± SEM. Compared with control: *P˂0.05, compared with BPA: **P˂0.05

Immunohistochemical examination of GFAP-stained cerebral cortex and hippocampus sections of both control and FA-treated rats showed weak positive immunoreactivity in fibrillary astrocyte body and processes (Fig. 7a, d, e &h). Meanwhile, GFAP-stained cerebral cortex and hippocampus sections of BPA-intoxicated rats revealed strong positive immunoreaction in the astrocyte body and processes that significantly increased by 18.3, 24 respectively, compared to the control group (Figs. 7b and 8a, b). On the other hand, cerebral cortex and hippocampus sections of BPA + FA administered rats revealed moderate GFAP immunoexpression in astrocyte body and processes that significantly decreased by 6.1, 7.5 respectively, compared to the BPA group (Figs. 7c, g and 8a, b).

### Molecular docking studies

**Fig. 9:**
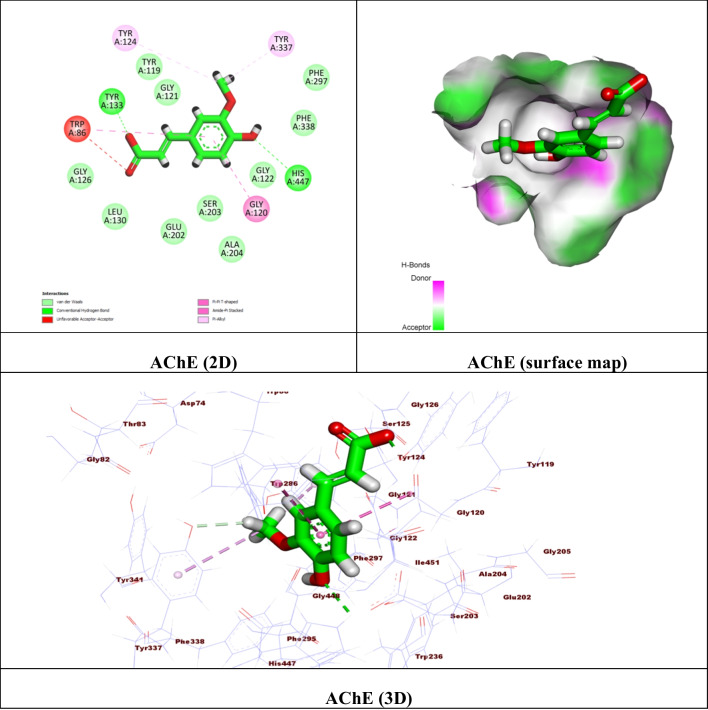
(**A**) 2D binding mode, (**B**) surface map, and (**C**) 3D binding mode of ferulic acid in the active site of the Human Acetylcholinesterase (AChE) (PDB ID: 4EY6)

The virtual docking studies of FA were carried out on three druggable proteins in treating Alzheimer’s disease. The compound showed remarkable binding affinities compared to the co-crystallized reference for the investigated proteins. Regarding AChE, the FA displayed a noticeable binding affinity with ΔG = -5.39 kcal/mol. Two hydrogen bonds were formed between oxygens of carboxylic and phenolic OH with the crucial amino acids Tyr 133 and His 447, respectively. Also, π-π interactions were presented between the phenyl ring of the compound with Gly 120 and Trp 86 (Fig. 9).

**Fig. 10:**
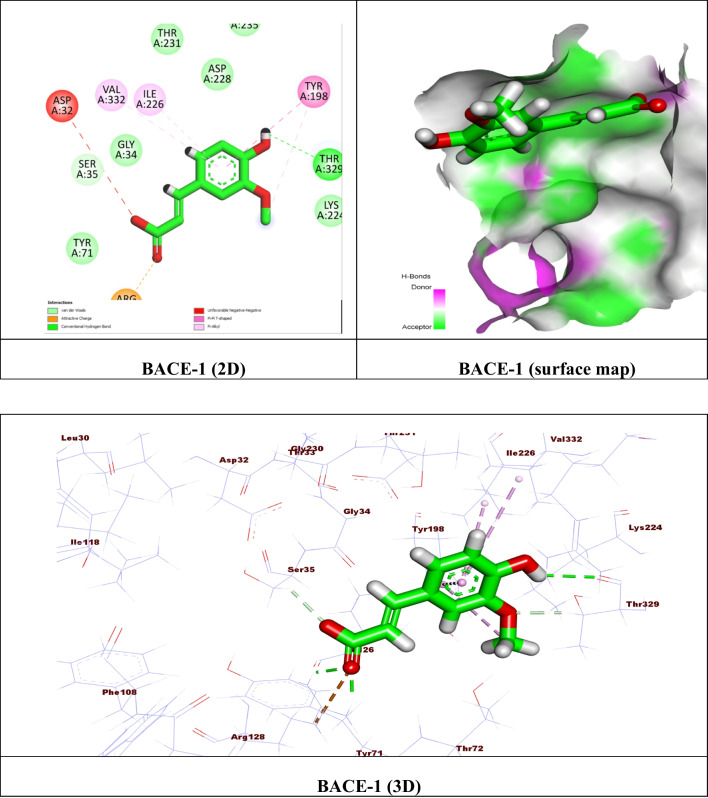
(**A**) 2D binding mode, (**B**) surface map, and (**C**) 3D binding mode of ferulic acid in the active site of Beta-Secretase 1 (BACE-1) (PDB ID: 7N66)

Similarly, toward BACE-1, the FA revealed moderate affinity with ΔG = − 4.60 kcal/mol, where hydrogen bonds were formed between the phenolic hydroxyl and the key amino acid Thr 329. Moreover, an attractive charge was shown between the carbonyl of carboxylic with Arg 128 (Fig.10).

**Fig. 11:**
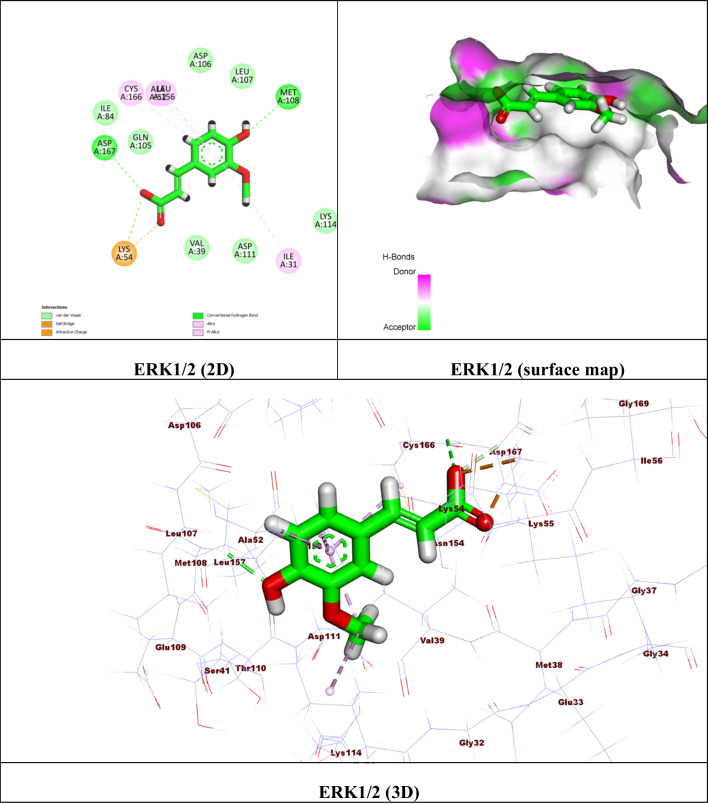
(**A**) 2D binding mode, (**B**) surface map, and (**C**) 3D binding mode of ferulic acid in the active site of the extracellular signal-regulated kinase 1/2 (ERK1/2) (PDB ID: 6G97)

The same with the ERK1/2, the compound exhibited good binding affinity with (ΔG = -5.39 kcal/mol). The study revealed the presence of 2 hydrogen bonds between the carboxylic and phenolic OH with the key amino acids Asp 167 and Met 108, respectively. Also, with the amino acid Lys 54, the carbonyl and OH of the carboxylic group formed two attractive charges (Fig. 11).

## Discussion

BPA is a well-known endocrine toxicant widely used in manufacturing plastics, dental adhesives, and thermal printing paper (Chen et al. 2017). BPA interferes with brain development and induces neurological diseases in vertebrates, such as diminished cognitive abilities and behavioral disorders (Santoro et al. 2019; Gyimah et al. 2021). However, exogenous nutritional interventions have been shown to mitigate the detrimental consequences of BPA (El Tabaa et al. 2017; Akintunde et al. 2020).

Our study is considered the first to investigate the neuroprotective effect of FA on BPA-induced AD-like pathology in male rats. Cognitive performance is evaluated using the Y-maze and NOR tests, which are behavioral assessments of spatial working memory and long-term memory, respectively. Herein, BPA-intoxicated rats exhibited a significant reduction in SAP in the Y-maze test, indicating a spatial memory impairment; these findings are consistent with the results of a previous investigation (El Tabaa et al. 2017). Conversely, treatment with FA could improve the spatial working memory deficit, as evidenced by an increment in SAP in rats co-administered BPA + FA. These results are in agreement with earlier reports (Mamiya et al. 2008; Jung et al. 2016; Khalifa et al. 2024).

Furthermore, results of the NOR test showed that rats exposed to BPA toxicity displayed a significant reduction in novel object preference % as well as a decline in discrimination ratio. These results are in line with previous studies (Eilam-Stock et al. 2012; El Tabaa et al. 2017; Morsy and Ahmed 2020). At the same time, rats treated with BPA + FA could reverse the deficits in NOR, visualized by significant increment in discrimination ratio and novel object preference%. These results are consistent with previous reports (Jung et al. 2016; Mori et al. 2017; Mhillaj et al. 2018). These neurobehavioral results indicated impairment in acquiring and retaining spatial memory and cognitive deficits, which resulted in learning and memory impairment (Barker and Warburton 2011).

BPA is a well-established neurodegenerative model that may be linked to oxidative stress development, as previous studies suggested (Babu et al. 2013; Alekhya Sita et al. 2019). where BPA exposure could liberate enzymatic and non-enzymatic radicles that react with NADPH or GSH; the latter reaction may raise both MDA and GSSG and deplete the GSH, causing oxidative stress (Espinosa-Diez et al. 2015). MDA is an oxidative stress marker indicating tissue lipid peroxidation (Zhang et al. 2017). Although GSH is the most abundant non-protein thiol, it is crucial for treating cerebrovascular illnesses, including AD, by protecting brain endothelial cells from oxidative stress (Song et al. 2014). According to a prior study, the stability of GSH and prevention of its oxidation make GSSG essential for intracellular antioxidant activity. Cellular health is indicated by the ratio of reduced GSH to oxidized GSH (GSSG) (James et al. 2009; Owen and Butterfield 2010).

In the present study, there were increments in both MDA and GSSG brain levels, indicating a state of oxidative stress, confirmed by the depletion of GSH content in BPA-intoxicated rats. However, rats treated with BPA + FA exhibited a significant decrease in MDA and GSSG and a significant increase in GSH levels in brain tissue. Hence, the administration of FA could ameliorate brain oxidative biomarkers. Several studies reported the antioxidant activities of FA in brain injury and cognitive deficits in the neurotoxicity model or AD-induced model by its ability to scavenge superoxide anion radical and inhibit lipid peroxidation (Ren et al. 2017; Yu et al. 2021; Park et al. 2022).

Oxidative stress and inflammation are the major hallmarks of AD. Once the antioxidant mechanism is depleted, protein aggregation and ROS generation occur, and mitochondria may act as a second messenger to activate the immune system. Consequently, increased proinflammatory cytokines (Simpson and Oliver 2020) and microglia immunological involvement are the main gate for neurodegeneration. Both microglia and TNF-α played mutual effectors in neuroprotection and neuroinflammation (Li and Barres 2018). Microglial activation by amyloid plaques induces pro-inflammatory responses, including IL-1β and TNF-α, which are key factors in the functional effects of neuroinflammation on neurodegeneration (Zhao et al. 2017).

The current data displayed a significant elevation of TNF-α and a non-significant elevation of IL-1β. Also, the previous reports stated the detrimental effects of BPA on the brain tissue of AD models and human macrophage cells; this toxic effect is expressed by elevated TNF-α and IL-1β (Chen et al. 2018; Sukjamnong et al. 2020; Engin and Engin 2021), thus may explain the neurodegenerative role of BPA. On the other hand, the administration of FA improved the brain levels of TNF-α and IL-1β suggesting its anti-inflammatory role where FA inhibited the activation of NLRP3 inflammasome that resulted in reduced transcriptional levels of IL-1β, IL-6, and TNF-α (Liu et al. 2022).

Furthermore, the Akt and ERK cascade is a potential targeted mechanism for neurodegenerative diseases. Akt, a serine/threonine kinase, plays a crucial role in cell survival, growth, and metabolism. However, recent research has demonstrated that p-Akt levels are elevated in the hippocampus and cortex of AD patients, suggesting a compensatory response to neuronal damage (Gao et al. 2022). ERK also controls cell proliferation, differentiation, and survival. Increased ERK activation has been observed in AD patients’ brains, particularly in areas with Aβ deposition. ERK may contribute to Aβ-induced neurotoxicity (Gao et al. 2022).

The existing study revealed the hyper-expressed level of ERK and p-Akt in BPA-intoxicated rats. Our findings are in agreement with the recent study that thoroughly discussed the toxicity effect of BPA on oxidative stress in rat brains (Kobayashi et al. 2020). Furthermore, inhibiting Akt activity reduces Aβ-induced neurotoxicity and enhances cognitive function in AD animal models (Ali et al. 2018). Similarly, reducing ERK activity can reduce Aβ-induced synaptic disruption, enhance memory, and offer a potential AD treatment target (Zeze et al. 2022). Consequently, it is notable that FA has significantly decreased ERK and p-Akt protein expression levels in conjunction with a work that described the molecular target of FA in attenuating focal cerebral ischemia injury by regulating ERK and p-Akt protein expression levels (Koh 2015).

Also, the formation of Aβ plaque, especially Aβ (1–42), is a crucial component of AD lesions (El-Hawary et al. 2021). The role of Aβ in surrounding microglia and initiation of neuroinflammatory degeneration and propagation of AD. The BPA-intoxicated group showed a significant increase in Aβ 1–42 compared with the normal control group. These results agree with earlier studies (Wang et al. 2017b). on the contrary, FA has significantly counteracted the deleterious effect of BPA by decreasing Aβ 1–42, as previously reported (Sultana et al. 2005). This effect may be explained by the remarkable binding affinity of FA with Beta-Secretase 1 (BACE-1), the enzyme responsible for the initial cleavage of the amyloid precursor into all monomeric forms of amyloid *β*. These results are confirmed by an in vitro analysis of the brain level of BACE that is significantly increased in BPA-intoxicated rats. While rats co-treated with BPA + FA showed considerable reduction in the BACE in comparison with intoxicated rats. The findings of our study are consistent with (Tripathi et al. 2020). These findings confirmed the neuroprotective impact of FA and established FA as a compelling treatment for AD.

Tau protein is a crucial component of neurofibrillary tangles, one of the hallmarks of AD (Muralidar et al. 2020). In addition, aggregation of the pTau leads to the instability of the cytoskeleton and subsequent neuronal dysfunction or death (Yan et al. 2018). The current study showed a significant increase in the tTau and pTau protein deposition in the BPA-intoxicated group. Our results are in agreement with earlier studies that reported that BPA exposure can elevate the levels of total and phosphorylated tau protein in the brain (Abdel-Rafei and Thabet 2020; Xue et al. 2020; Flores et al. 2022). Previous research showed that FA reduces tau protein and improves cognitive impairment in rats (Wang et al. 2017b). Moreover, the docking data revealed FA’s promising binding affinities with ERK 1/2 kinases, which contribute to the formation of pathological tau conformation.

Furthermore, the findings of our investigation indicated that the BPA-intoxicated group revealed a significant increase in the AChE brain levels compared to the control group. However, rats co-administrated with BPA + FA showed a significant decline in AChE levels compared to intoxicated rats. Our results agreed with (Tripathi et al. 2020), who recorded the inhibitory effect of FA on the AChE activity. Also, molecular docking studies reported that FA has good binding affinities toward AChE, and these results make FA a promising therapy for AD.

In the current study, the cerebral cortex and hippocampus sections of rats intoxicated with BPA showed several histopathological alterations. Cognitive ability disorders are linked to damaged neuronal cells in the hippocampus, one of the most important learning regions. The cerebral cortex showed pyknotic and degenerated neurons with pericellular spaces and neuropil vacuolation. The hippocampus revealed pyknotic neuroglia with perineural space in molecular and polymorphic layers. The pyramidal cell layer exhibited disarrangement of pyramidal neurons; some neurons appeared pyknotic and degenerated with pericellular spaces, and some neurons had nuclei with chromatolysis. There was neuropil vacuolation. These findings agreed with Abd Elaziz and Laag (2018), who reported that rats administered BPA had a hippocampus with disorganized, degenerated pyramidal cells that lost their normal arrangement and appeared shrunken with pericellular spaces. Disarrangement of pyramidal cells may be atrial to regain function (Abd Elaziz and Laag 2018). Altshuler et al. (1987) stated that the appearance of dark neurons with condensed cytoplasm and nucleoplasm may contribute to apoptosis. Neuropil vacuolation may revert to the shrinkage of cells and withdrawal of their process secondary to cytoskeletal fragmentation, thereby leaving pericellular spaces (Galal et al. 2019). Ratan et al. (1994) stated that BPA can lead to oxidative stress and DNA damage. Consequently, free radicals provoked by oxidative stress can cause neural degeneration.

On the other hand, cerebral cortex and hippocampus sections obtained from rats cotreated by BPA + FA revealed marked recovery compared to the BPA group in the form of maintenance of the normal architecture of some pyramidal neurons that had vesicular nuclei with clear nucleoli in the cerebral cortex and maintenance of nearly normal architecture of the three layers of the hippocampus with a normal linear arrangement of pyramidal nerve cells. This ameliorative effect of ferulic acid may be attributed to its antioxidant role, as it is one of the phenolic chemicals found in Pumpkin seed oil (PSO) (Sirasanagandla et al. 2022). Fawzy et al. (2018) supported our results and reported that PSO supplementation decreased DNA damage and enhanced histopathological changes in the liver and testes tissues exposed to BPA.

GFAP is an intermediate filament protein present in the neuroglia of the CNS, including astrocytes (Sobaniec-Lotowska 2001). Also, GFAP is considered a specific marker for the maturity of astrocytes in the CNS (Eng et al. 2000). In the present research, GFAP immunoexpression was significantly positive in the cerebral cortex and hippocampus of rats intoxicated with BPA compared to control rats. This result correlated with Abd Elaziz and Laag (2018), who reported a significant increase in GFAP immunoreaction in the hippocampus of rats that received BPA intraperitoneally. This astrogliosis may be a compensatory mechanism for repair against neuronal damage (Abd Elaziz and Laag 2018). Meanwhile, administering BPA + FA significantly reduced GFAP immunoexpression in the cerebral cortex and hippocampus compared to the BPA group. This finding refers to the powerful antioxidant effect of FA that aligns with Ren et al. (2017), who reported that FA is regarded as a free radical scavenger with antioxidant and anti-inflammatory effects in the brain.

In conclusion, FA exhibits potent antioxidative and antiapoptotic activities, as demonstrated by its ability to ameliorate cognitive impairment, decrease brain biomarker levels, and restore normal brain architecture in BPA-induced AD-like pathology in male rats.

## Electronic supplementary material

Below is the link to the electronic supplementary material.

Supplementary Material 1 (DOCX 67.4 KB)
