# Supplementary material for: Ferulic acid ameliorates bisphenol A (BPA)-induced Alzheimer’s disease-like pathology through Akt-ERK crosstalk pathway in male rats
Source: Psychopharmacology (Berl). 2024 Oct 23;242(3):461–80. doi: 10.1007/s00213-024-06697-4 (PMC11861243; doi:10.1007/s00213-024-06697-4)
Supplement: Supplementary file 1 — Supplementary Material 1 (DOCX 67.4 KB) [file 213_2024_6697_MOESM1_ESM.docx]

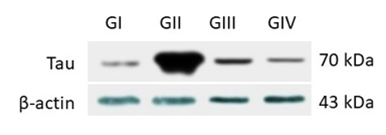


**Supplementary figure 1.** Effect of BPA administration for 40 days on the brain level of the total Tau protein expression level. Group Ⅰ (control group), Group Ⅱ (BPA-intoxicated rats), Group Ⅲ (rats co-administered BPA+FA), and Group Ⅳ (FA-treated rats).
